# IMPROVING COGNITIVE FUNCTION IN THE NEW COHORT—BUT ONLY FOR MEN. FINDINGS FROM THE KYOTANGO CENTENARIAN STUDY

**DOI:** 10.1093/geroni/igad104.1338

**Published:** 2023-12-21

**Authors:** Yasuyuki Gondo, Xinyu Zhang

**Affiliations:** Osaka University, Suita, Osaka, Japan; Osaka University, Suita, Osaka, Japan

## Abstract

The Kyotango Centenarian Cohort Study was established with the aim of tracing long-term changes in the overall functioning of centenarians. This study started in 2014 in Kyotango City. Since then, new centenarians and those who were expected to be 100 years old the following April were invited to the study every year in September. Eligible centenarians were invited by the city, and centenarians and their families agreed to participate to answer questions either by mail or face-to-face interviews, according to their preference. For the non-participants, anonymized living conditions and care need levels were provided by the city. The number of centenarian candidates varied by year (24-55). However, the number of woman centenarians had increased through the 8-year surveyed period (31 and 42 for women, 10 and 9 for men in 2014 and 2021). On average, every year 40 new centenarians were invited and 80% of them participated in the survey. Among the functional variables, cognitive function was estimated by proxies, which had a high correlation with MMSE. No clear improving trend for nursing care level in Japanese long-term care insurance, as measured by activities of daily living and cognitive function rating, was observed. Meanwhile, an improving trend for cognitive function was observed among men but not among women. This might be caused by the gender difference in the survival ratio that frail women were more likely to survive. Future research should investigate possible reasons for a cohort improvement in Japanese centenarian men, and cognitive intervention programs should particularly target women.

